# Boys Don't Cry: Examining Sex Disparities in Behavioral Oncology Referral Rates for AYA Cancer Patients

**DOI:** 10.3389/fpsyg.2022.826408

**Published:** 2022-02-17

**Authors:** Martin Kivlighan, Joel Bricker, Arwa Aburizik

**Affiliations:** ^1^Department of Psychological and Quantitative Foundations, College of Education, The University of Iowa, Iowa City, IA, United States; ^2^Department of Psychiatry, Carver College of Medicine, The University of Iowa, Iowa City, IA, United States; ^3^Department of Internal Medicine (Hematology-Oncology), Carver College of Medicine, The University of Iowa, Iowa City, IA, United States

**Keywords:** adolescents and young adults (AYA), access, sex disparities, psychosocial distress, psycho oncology

## Abstract

Psychosocial distress is highly prevalent in cancer patients, approaching rates around 40% across various cancer sites according to multicenter studies. As such, distress screening procedures have been developed and implemented to identify and respond to cancer patients' psychosocial distress and concerns. However, many cancer patients continue to report unmet psychosocial needs suggesting gaps in connecting patients with psychosocial services. Presently, there is a paucity of research examining sex-based disparities in referral rates to behavioral oncology services, particularly for adolescent and young adult (AYA) cancer patients. Informed by gender role conflict and empirical literature documenting disparities in cancer care and treatment based on a variety of sociocultural variables, this study aimed to examine the presence of sex disparities in referral rates to behavioral oncology services for AYA cancer patients. Data for this study consisted of 1,700 AYA cancer patients (age 18–39) who completed a distress screening at a large cancer center of a teaching hospital in the Midwestern United. Results indicated that patient sex significantly predicted the odds of behavioral oncology referral (γ_50_ = −0.95, Odds ratio = 2.60, *p* < 0.001). This finding indicates that female AYA cancer patients are 2.5 times more likely to be referred to behavioral oncology services compared to male AYA cancer patients after controlling for psychosocial distress and emotional, family, and practical problems. Additionally, we found that emotional problems significantly moderated the odds of referral for males and females (γ_60_ = 0.37, Odds ratio = 1.44, *p* < 0.001), however the odds of referral for males who endorsed emotional problems were lower than males who did not endorse emotional problems. This contrasted with female AYA cancer patients where the endorsement of emotional problems increased the odds of referral to behavioral oncology services. Findings are discussed with particular focus on how to enhance equitable care and reduce sex and other sociocultural-based disparities in AYA psychosocial oncology.

## Introduction

Psychosocial distress is highly prevalent in cancer patients, approaching rates between 40 and 60% across various cancer sites according to multicenter studies (Zabora et al., 2001). In a large multicenter study consisting of 55 cancer treatment centers in the United States and Canada, 46% of patients reported significant distress as measured by the Distress Thermometer (Carlson et al., 2019; Essue et al., 2020). In another meta-analysis, prevalence rates of depression, anxiety, and adjustment disorder in cancer patients were 16.5, 9.8, and 15.4%, respectively (Mitchell et al., 2011). Unabated, psychosocial distress in cancer patients has negative sequalae on quality of life, emotional wellbeing, psychosocial functioning, access and adherence to cancer care, and biological outcomes (IOM, 2007; Caruso and Breitbart, 2020). Additionally, unresolved psychosocial problems in cancer patients have adverse economic sequalae on the patient and health care system (Cardoso et al., 2013; Doherty et al., 2019). These are exemplified by longer hospital stays, lack of adherence to scheduled visits and prescribed treatments, increased unplanned visits to emergency rooms with additional imaging and work-up, amongst other avoidable expenses (Carlson and Bultz, 2004; Essue et al., 2020).

These adverse effects prompted national standards of cancer care to include identifying and addressing psychosocial needs of cancer patients as an integral part of cancer care (Jacobsen and Wagner, 2012). Indeed, several agencies, including the American Society of Clinical Oncology, the Canadian Association of Behavioral oncology, and the Institute of Medicine have developed guidelines and recommendations for the implementation of systematic identification of psychosocial distress in cancer patients (Carlson and Bultz, 2003; IOM, 2007). Distress screening processes were developed utilizing validated instruments and tools to identify and assess the severity of psychological distress in cancer patients coupled with recommendations for triage processes to ensure interventions and follow up are implemented (Loscalzo et al., 2013; Donovan et al., 2014; Bultz, 2017). Despite these advancements in the practice of identifying and treating psychosocial distress, important limitations exist (Carlson, 2013; Bultz et al., 2021) and many cancer patients continue to report unmet psychosocial needs (McMurtry and Bultz, 2005; Meggiolaro et al., 2021). This is partly attributable to ineffective psychosocial distress screening procedures, patients' help-seeking behaviors, limited access or availability of psychosocial services, as well as provider attitude and cultural factors (Dilworth et al., 2014; Brebach et al., 2016; Carolan et al., 2018). The discrepancy between heightened psychosocial needs and the bridging of patients with psychosocial services is especially pronounced in some minority groups and vulnerable populations who bear the brunt of psychosocial adversity collateral to the physical impact of a diagnosis cancer (Kamen et al., 2017; Kent et al., 2019).

One understudied barrier to behavioral oncology care is gender/sex bias and disparities. A large body of research exists documenting disparities in oncology treatment and care based on various sociocultural identities, such as race (Emerson et al., 2020; Hardy and Du, 2021), socioeconomic status (SES) (Dreyer et al., 2018; Karanth et al., 2019), and gender (Tabaac et al., 2018; Benchetrit et al., 2019) however, less attention has been paid to sociocultural disparities, namely gender/sex disparities, in referral practices to behavioral oncology services. Given previous research documenting disparities in cancer care, and the need to increase utilization rates of behavioral oncology for cancer patients with psychosocial distress, this study aims to examine the presence of sex disparities in referral rates to psychosocial services (i.e., counseling and psychiatric services) for Adolescent and Young Adult (AYA) cancer patients.

### AYA Psychosocial Needs

AYA cancer patients are recognized as an underserved minority within cancer patients whose unique developmental and social attributes put them at a particular disadvantage related to social, interpersonal, academic, occupational, and financial sequalae of a cancer diagnosis further deepening the chasm in cancer care between them and their non-AYA counterparts (Clinton-McHarg et al., 2010; Jacobs et al., 2018).

Longitudinal data demonstrates patterns of disproportionate and lasting financial burden, consequences of interrupted education and work, difficulties with relationships and family planning, and unresolved physical and mental health issues related to AYA history of cancer (Smith et al., 2019). Evidence also suggests that the mere availability of psychosocial services for AYA patients is not a factor in reducing and preventing future psychosocial dysfunction in this patient population (Patterson et al., 2017; Jacobs et al., 2018) calling for a higher level of communication and triage of these patients to psychosocial services. Ongoing efforts include thoughtful evaluation of age-specific screening tools for identifying psychosocial distress in AYA, strategies honing in on areas of success and where improvement is needed to produce interventions that are specifically tailored to this age group.

### Barriers to Behavioral Oncology

Despite effective screening tools and the proliferation of evidence-based care for cancer patients, problems remain in the equity of service delivery and referral processes. Psychosocial oncology literature has demonstrated that disparities exist in psychological distress screening, referral of patients to psychosocial resources, and the utilization of such resources by patients (Kamen et al., 2017; Nolan et al., 2018). Barriers to access psychosocial services are multifaceted and include systemic, patient, and provider factors, such as low referral rates by physicians, patients' perceived stigma about accessing mental health services, and fragmentation of care (Matthews et al., 2004). For example, Dilworth et al. (2014) conducted a systematic review and found that physicians' negative perceptions about psychosocial services were one of the most common barriers to care. These negative perceptions stemmed from the perceived lack of evidence-backed research for psychosocial interventions, the potential to cause psychosocial harm, and the priority to control cancer-related symptoms over psychosocial care (Dilworth et al., 2014). In another study, patient age was found to significantly contribute to the likelihood of referral to psychosocial care in a sample of metastatic cancer patients, such that younger patients were more likely to be referred to psychosocial services (Ellis et al., 2009). Regarding utilization rates of psychosocial services, one study found that the majority of cancer patients attending counseling services were well-educated, urban-residing women (Nekolaichuk et al., 2011). Together, this body of research suggests that some cancer patients may be more or less likely to be referred to psychosocial services based on demographic factors, such as age, education, and SES. Regarding research that has found that on average, women cancer patients utilize psychosocial services at greater rates compared to men, it may be that women are more likely referred to these services compared to men resulting in differential utilization rates. Gender role conflict and social constraint may explain this phenomenon, and support research examining the presence of gender disparities in behavioral oncology referral rates (Strong et al., 2007; Salk et al., 2017).

### Gender Role Conflict

Gender role conflict (GRC) and traditional masculinity norms are important factors that can lead to compromised adjustment in men with cancer (Nicholas, 2000). As noted by O'Neil et al. GRC refers to the negative cognitive, emotional, and behavioral consequences associated with male socialization (O'Neil et al., 1986). Specifically, O'Neil defined GRC as “a psychological state in which gender roles have negative consequences or impact on the individual or on others” (p. 25) (O'Neil, 1990). Within a cancer diagnosis context, research has demonstrated how traditional and restrictive masculinity and gender role conflict are related to poorer physical and psychological outcomes in men with cancer (Maliski et al., 2008; Hoyt, 2009). In one study, cancer-related masculine threat was associated with poorer physical outcomes over time in a sample of men with prostate cancer (Hoyt et al., 2013).

One theorized mechanism through which gender role conflict may negatively influence physical and psychological outcomes of men with cancer is emotional approach coping (Lennon et al., 2018). Emotional approach coping has been defined as identifying, understanding, and expressing emotions appropriately and is posited to consist of two strategies, emotional processing, and emotional expression (Lennon et al., 2018). Interestingly, Hoyt et al. (2013) also found that cancer-related masculine threat was significantly associated with decreased emotional processing, which ultimately explained the effect of cancer-related masculine threat on poor physical outcomes. In another study, gender role conflict was found to significantly predict distress in a sample of men with prostate cancer (Lennon et al., 2018). Together, these findings suggest that gender role conflict and emotional approach coping, or the tendency for men to restrict emotional expression, may contribute to negative cancer-related physical and psychological outcomes. Simultaneously, gender role conflict may also impact the likelihood that providers and care team members will refer men to behavioral oncology services as talking about emotional distress with men may violate traditional male gender role socialization (Vogel et al., 2014).

To our knowledge, no study has examined the presences of sex disparities in referral rates to behavioral oncology services, nor has this question been studied in the AYA population. This is important given literature suggesting that younger adult males tend to experience more gender role conflict than older adult males (Watts and Borders, 2005). One study found that young adult men who reported greater restrictive emotionality endorsed lower levels of resiliency in the face of adverse experiences (Galligan et al., 2010). In another study, Pederson and Vogel (2007) examined several mediators of the relationship between gender role conflict and college-aged men's willingness to seek counseling. Results indicated that men who experienced greater gender role conflict were less likely to disclose distressing information, which subsequently led to less positive attitudes and willingness to seek counseling. These studies are important as they highlight the fact that gender role conflict occurs across the lifespan and may have unique consequences particularly for AYA men.

### Purpose of Study

This study examines the presence of sex-based disparities in referrals to behavioral oncology within an AYA cancer patient population. Research has documented the importance of screening and responding to cancer patients' psychosocial distress. However, research has demonstrated how distress screening and referral efforts may not be equitable across all cancer patients. Yet, few studies have examined sociocultural barriers to access for AYA cancer patients. Particularly, there is a paucity of research examining if referral rates to behavioral oncology services vary based on patients' sex in AYA cancer patient populations. Informed by gender role conflict and empirical literature documenting disparities in cancer are and treatment based on a variety of patient demographic variables, this study aimed to examine the presence of sex disparities in referral rates to behavioral oncology services for AYA cancer patients, including individual counseling or psychiatric services. It is important to note that sex and gender represent distinct constructs and should not be used interchangeably. Gender is the range of characteristics pertaining to, and differentiating between femininity and masculinity, whereas sex refers to an individual's biological makeup resulting in a male or female phenotype. While the spectrum of gender identities (and even biological sex) is wide, this study focuses specifically on sex disparities (i.e., male and female) due to the data available in our archival data set which was limited to the patients' reported sex, rather than their gender identity. However, despite this limitation, we believe that examining sex-based disparities informed by gender role conflict is an important endeavor in the field of psychosocial oncology. Given the aforementioned literature on the role of gender role conflict on cancer related outcomes for men, we proposed the following hypothesis.

*Hypothesis 1*: Sex disparities will exist in behavioral oncology referrals, such that male AYA cancer patients will have lower odds of being referred to behavioral oncology services compared to their female AYA cancer patient counterparts.

As a second aim, we sought to examine male AYA cancer patients' endorsement of emotional problems on the likelihood that they would be referred to behavioral oncology services. Emotional approach coping is theorized to serve as an important factor in male cancer patients' health outcomes, as well as their willingness to seek treatment. It may be that male AYA cancer patients are less likely to be referred to behavioral oncology services because they are less likely to disclose distressing information as a result of emotional approach coping (Pederson and Vogel, 2007). Informed by the theory of emotional approach coping we proposed the following hypothesis.

*Hypothesis 2*: The odds of male AYA cancer patients being referred to behavioral oncology care will significantly vary as a function of their endorsement of emotional problems, wherein male AYA cancer patients who endorse emotional problems will be more likely to be referred to services compared to male AYA patients that do not endorse emotional problems.

## Methods

### Participants

The data for this study consisted of 1,700 AYA patients (age 18–39) with a diagnosis of cancer at a large cancer center of a teaching hospital in the Midwestern United States. Patient characteristics are reported in Table 1. The average age was 30.38 (SD = 6.08) with a range of 18–39 years old. 70.4% of the sample were female (*n* = 1,197) and 29.6% were male (*n* = 503). Regarding race/ethnicity, 83% (*n* = 1,411) patients identified as white, 8.2% (*n* = 139) identified as Black/African American 2.8% (*n* = 48) identified as Hispanic/Latino/a, 2.7% (*n* = 46) identified as Asian/Asian American, 1.4% (*n* = 24) identified as Multiracial, <1% (*n* = 6) American Indian/Alaska Native, <1% (*n* = 2) identified as Native Hawaiian/Pacific Islander, and 1.5% (*n* = 24) did not report their race/ethnicity. Regarding oncology department, 1,076 (63.3%) patients were seen in hematology oncology, 82 (4.8%) in pulmonary oncology, 348 (20.5%) in gynecology oncology, 149 (8.8%) in surgical oncology, and 45 (2.6%) in urology oncology. The 1,700 patients were seen by 51 nurses. No demographic data for oncology providers or nurses was available in the data set.

**Table 1:** Patient characteristics.

**Characteristics**	***n* (%)**
Age, mean (SD)	30.38 (6.08)
Male	503 (29.6)
Female	1,197 (70.4)
**Race/ethnicity**	–
White	1,411 (83.0)
Black/African American	139 (8.2)
Hispanic/Latino/a	48 (2.8)
Asian/Asian American	46 (2.7)
Multiracial	24 (1.4)
American Indian/Alaska Native	6 (<1.0)
Native Hawaiian/Pacific Islander	2 (<1.0)
**Department**	–
Hematology Oncology	1,076 (63.3)
Pulmonary Oncology	82 (4.8)
Gynecology Oncology	348 (20.5)
Surgical Oncology	149 (8.8)
Urology Oncology	45 (2.6)

### Measures

#### Distress Screening Questionnaire

The distress screening questionnaire is an adapted version of the NCCN distress thermometer (DT) which consists of the single question rating the level of distress of patients over the week prior to their visit, associated with a problem list requiring yes/no answers to the presence of difficulties in three domains: practical problems (i.e., financial, transportation, insurance, etc.), family problems (i.e., problems with partner, siblings), and emotional problems (i.e., depressive, anxiety, sleep problems as well as existential questions and ambivalence about spirituality). The distress thermometer is a one item visual analog that assesses individual's distress level from 1 to 10, where 1 is low distress and 10 is high distress.

#### Behavioral Oncology Referral

Referral rates to behavioral oncology services were accessed through patient medical records. Specifically, a medical record review allowed the research team to identify which patients had an order placed for behavioral oncology and which patients did not (i.e., referral vs. no referral). Behavioral oncology services consisted of either individual counseling or psychiatric care. Referral to behavioral oncology services was dummy coded (0 = no referral, 1 = referral).

### Procedures

At the participating cancer center all new cancer patients are assigned a distress screening questionnaire prior to their first appointment, and every 3 months upon follow up, to assess psychosocial distress and concerns. The distress screening questionnaire consists of the distress thermometer and three yes/no questions assessing the presence of practical problems (i.e., financial, transportation, insurance, etc.), family problems, and emotional problems. Archival data for this study was accessed through the electronic medical record system of the participating site. Archival data included patient demographic data, appointment data, distress screening data, including patient distress scores and problems indicated and order status for behavioral oncology (i.e., referral placed). Behavioral oncology services consisted of individual counseling services with a licensed mental health provider or psychiatric services with a psychiatry provider. All procedures were approved by the first author's institutional review board.

### Data Analysis Plan

Data was analyzed using multilevel logistic modeling to account for the nested nature of our data (i.e., patients nested within nurses) and examine the odds of referral to behavioral oncology services for male identified and female identified AYA cancer patients. Specifically, we used Hierarchal Linear Modeling (HLM; Raudenbush et al., 2011) to run a 2-level model with patient sex (0 = male, 1 = female) as a level-1 predictor of referral to behavioral oncology (0 = no referral, 1 = referral). Patients' distress thermometer score, and presence of emotional, family, and practical problems (0 = no, 1= yes) were entered as covariates at level 1. In addition to modeling fixed effects of the overall odds of referral based on patients' sex, we examined the variability in the odds of referral based on patient sex between nurses at level 2. Specifically, we included a random component at level 2 to examine between-nurse variability in the odds of male and female AYA cancer patients being referred to behavioral oncology. To test our second hypothesis, we included an interaction term between emotional problems and patient sex in our model. This interaction term tested whether the odds of being referred to behavioral oncology varied as a function of male AYA cancer patients' endorsement of emotional problems.

## Results

Overall, the average distress score was 3.38 (SD = 2.82), with an average score of 2.90 (SD = 2.74) for males and 3.59 (SD = 2.84) for females. On average, 40.0% of patients endorsed practical problems, 18% endorsed family problems, and 45% endorsed emotional problems, with 41, 12, and 38% of males endorsing practical, family, and emotional problems, respectively, and 40, 20, and 48% of females endorsing practical, family, and emotional problems, respectively.

Results from the multilevel logistic analysis of patient sex on the odds of behavioral oncology referral are reported in Table 2. Our first hypothesis that—male AYA cancer patients will have lower odds of being referred to behavioral oncology services compared to their female AYA cancer patient counterparts—was supported. Specifically, patient sex significantly predicted the odds of behavioral oncology referral (γ_50_ = 0.95, Odds ratio = 2.60, *p* < 0.001). This finding indicates that female AYA cancer patients were ~2.5 times more likely to be referred to behavioral oncology services compared to male AYA cancer patients after controlling for distress levels and emotional, family, and practical problems. All random components at level 2 were not significant.

**Table 2:** Multilevel logistics model of sex disparities in behavioral oncology referrals.

**Variable**	**Estimate**	***SE***	**Odds ratio**	***df***	***p*-value**
Behavioral oncology referral, *γ_00_*	−3.05	0.06	0.05	50	< 0.001
Distress thermometer, *γ_10_*	0.02	0.01	1.02	50	0.060
Practical problems, *γ_20_*	−0.11	0.03	0.90	50	0.003
Family problems, *γ_30_*	0.17	0.05	1.19	50	< 0.001
Emotional problems, *γ_40_*	−0.04	0.03	0.96	50	0.175
Patient sex, *γ_50_*	0.95	0.07	2.60	50	< 0.001

**Figure 1:**
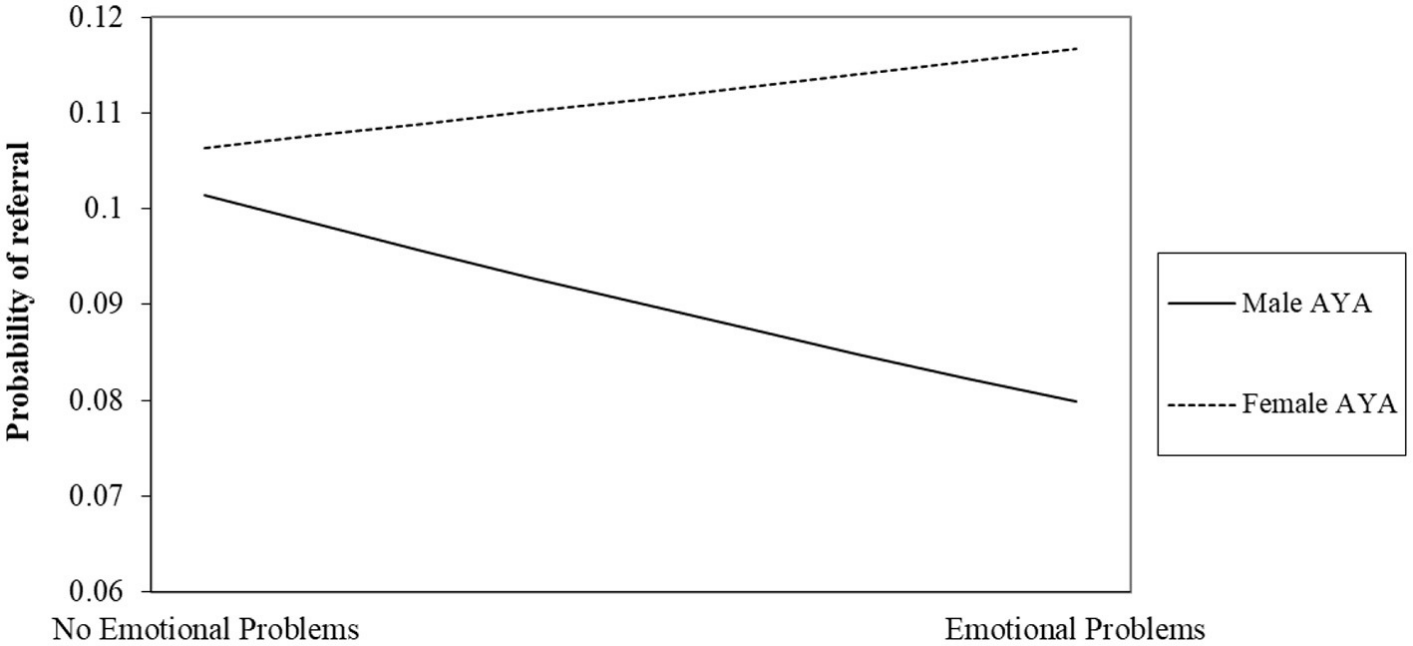
Interaction between emotional problems and sex on odds of referral to behavioral oncology.

Our second hypothesis that—the odds of male AYA cancer patients being referred to behavioral oncology care will significantly vary as a function of their endorsement of emotional problems, wherein male AYA cancer patients who endorse emotional problems will be more likely to be referred to services compared to male AYA patients that do not endorse emotional problems—was supported, but in the opposite direction. Specifically, we found that emotional problems significantly moderated the odds of referral for males and females (γ_60_ = 0.37, Odds ratio = 1.44, *p* < 0.001), however the odds of referral for males who endorsed emotional problems were lower than males who did not endorse emotional problems. As seen in Figure 1, this contrasts with female identified AYA cancer patients where the endorsement of emotional problems increased the odds of referral to behavioral oncology services.

## Discussion

This is one of the first studies to examine the presence of sex disparities in access to behavioral oncology services for AYA cancer patients. As hypothesized, our results indicated that sex disparities in behavioral oncology referral rates exist for AYA cancer patients, such that female AYA cancer patients have significantly higher odds of referral to behavioral oncology compared to their male AYA counterparts. This study is meaningful as it adds to the limited literature on sociocultural disparities in behavioral oncology for AYA cancer patients who are disproportionately impacted by adverse psychosocial sequalae of cancer (Nass et al., 2015; Smith et al., 2019). These findings support previous research that has documented disparities in psychosocial care and inequity of service delivery and referral processes to behavioral oncology services. As referenced earlier, a burgeoning body of research has documented unequitable practices in distress screening, referral of patients to psychosocial resources, and the utilization of such resources by patients (Ellis et al., 2009; Nekolaichuk et al., 2011; Kamen et al., 2017; Nolan et al., 2018).

Interestingly, our second finding indicated that endorsing emotional problems on the distress screening increased the odds of referral to behavioral oncology for female AYA patients but decreased the odds of referral for male AYA patients. Although we were unable to disentangle provider and patient factors that may contribute to sex disparities in accessing behavioral oncology services, this finding suggests that even when male AYA patients do not endorse traditional gender roles and disclose emotional problems, they are less likely to be referred to behavioral oncology care. It may be that despite male AYA patients' ability to disclose emotional distress, providers overlook these concerns related to their own gender role socialization. Acknowledging and discussing emotional concerns with men is often proscribed in the US, and therefore, providers acknowledging and further assessing male AYA cancer patients' emotional concerns would go against these gendered social norms. However, it is important to note that this explanation of our findings is post hoc, and future research is needed to understand this finding and possible explanation further.

Access to psychosocial services is influenced by system, provider, and patient factors (Matthews et al., 2004) and a large body of research has documented disparities in oncology treatment and care based on various sociocultural identities, such as race (Emerson et al., 2020; Hardy and Du, 2021), SES (Dreyer et al., 2018; Karanth et al., 2019), and gender (Tabaac et al., 2018; Benchetrit et al., 2019). Access may also be subject to provider biases based on attitudes, socialized norms, as well as patients' perceived stigma around mental health services and varying communication styles and willingness to disclose emotional and psychosocial distress.

### Implications for Practice and Future Research

There are several implications for AYA behavioral oncology care given the findings of this study. First, disparities in referral rates between male and female AYA patients maybe partially related to gender role conflict, which posits men are socialized to avoid emotions and behaviors that are considered to be less masculine. These behaviors may include discussing one's emotional symptoms or outwardly expressing one's inner emotional state. As such, it may prove beneficial to intervene with male AYA cancer patients to examine and address socialized gendered behaviors and attitudes about help-seeking to increase their willingness to disclose distressing experiencing with their care team and seek services when available. This may include psychoeducation for AYA cancer patients on the prevalence of psychosocial concerns within this population, the availability of behavioral oncology services, and the effectiveness of these services. Additional interventions can include narrative based health messaging that highlights the experiences of other male AYA cancer patients disclosing their emotional concerns and their experiences of seeking help.

A second factor that may explain sex disparities in access to behavioral oncology is provider attitudes and socialized gender roles. As discussed above, providers and other oncology staff may be less likely to offer a referral to behavioral oncology services if providers themselves have been socialized to avoid discussing emotions related to cancer with their male patients for fear of undermining masculinity (Vogel et al., 2014). As such, interventions aimed at exploring providers socialized gendered expectations and attitudes toward mental health and help seeking may be effective to increase awareness of potential biases. These may include provider education about the cost of unresolved psychosocial adversity in males and females alike, and the impact of dismissing male psychosocial distress and the prevalence of working-age men with cancer who go without having their psychiatric problems treated due to a variety of factors (Akechi et al., 2020). These interventions may help to reduce provider bias and increase their willingness to acknowledge, explore, and validate emotional concerns and refer to appropriate behavioral oncology services for all AYA cancer patients, including men.

Additional studies examining patient and provider factors of gender/sex disparities in access to behavioral oncology care are needed. Specifically, studies examining patient and provider attitudes about mental health and help-seeking may prove helpful for enhancing our understanding of critical factors to address in increasing access to services for all AYA cancer patients. Tools such as the Gender Role Conflict Scale (GRCS) and its short form could be used to examine both provider and patient gender role conflict and their willingness to disclose and explore psychosocial functioning and distress as well as the likelihood of referral and treatment seeking (O'Neil et al., 1986; O'Neil, 2015). Related, studies can examine the interactions/communication between patients and providers that may contribute to help-seeking. Advances in machine learning and natural language processing technologies may make this possible and could assist in identifying important factors within the patient-provider interaction that could increase access to behavioral oncology care. Lastly, research will need to ultimately test the effectiveness of interventions aimed at increasing access to behavioral oncology services, especially for male AYA cancer patients. Beyond identifying factors contributing to gendered disparities in accessing behavioral oncology care, research is needed to determine if these factors are malleable and if which interventions are effective in addressing these disparities and increasing access.

### Limitations

With any study, there are limitations. One limitation of this study is the use of a single site to examine disparities in access to behavioral oncology care. Although we used a relatively large sample, our findings may be related to site-specific phenomenon and factors and may not generalize to other cancer centers and systems. Future research can address this by replicating these findings with a multi-center design and larger sample size. Another limitation is that our study used archival data obtained from an electronic medical record (EMR) database and are therefore subject to data quality and accuracy issues often present when using EMR data for research. Additionally, sex was obtained from the EMR and may be limited in comparison to patient's gender. For example, patients' gender identity and expression may differ from their sex assigned at birth and sex was defined as a binary construct (Freiburger, 2018). We believe this not only represents an important limitation of our study, but the larger healthcare system as a whole. Moving forward, healthcare and EMR systems could allow patients to record their sex and gender outside of a binary construct as an affirming practice for patients with diverse gender identities. Related, our findings are only generalizable to male and female AYA patients, and future research is needed to explore these findings regarding gendered disparities to psychosocial services, particularly for gender minority patients. Another limitation is that we do not know what occurred in the appointments with patients and providers, which is important data that may help to further explain the presence of sex disparities. Finally, referrals to behavioral oncology services were recorded as a consultation order for behavioral oncology within the EMR, which would not have captured patients who were referred to outside providers, or those who were already receiving care for psychosocial needs through services outside of our site (i.e., community-based mental health centers).

## Conclusion

Disparities in psychosocial services for AYA cancer patients mirror inequitable services seen in other disciplines of medicine and have pervasive consequences for patients. These disparities can stem from systemic, provider and patient factors, and can further exacerbate poor psychosocial and health outcomes. Interventions highlighting the value and availability of psychosocial services to AYA patients, minimizing stigma around mental health, and addressing unconscious bias is imperative to foster equity in the access to psychosocial services. It is our hope that this study sheds light on the prevalence of gendered disparities in access to behavioral oncology for male AYA cancer patients and encourages future research to address inequities in access to care.

## Data Availability Statement

The raw data supporting the conclusions of this article will be made available by the authors, without undue reservation.

## Ethics Statement

The studies involving human participants were reviewed and approved by University of Iowa Internal Review Board. Written informed consent for participation was not required for this study in accordance with the national legislation and the institutional requirements.

## Author Contributions

AA provided oversight, supervision of research project and team, conceptualized the study, managed data, wrote manuscript, and prepared for submission. MK conceptualized study, analyzed data, wrote manuscript, and prepared for submission. JB wrote manuscript and prepared for submission. All authors contributed to the article and approved the submitted version.

## Funding

Funds for open access publication fees were received from the Division of Hematology, Oncology, and Bone Marrow Transplant in the Department of Internal Medicine at the University of Iowa Hospitals and Clinics.

## Conflict of Interest

The authors declare that the research was conducted in the absence of any commercial or financial relationships that could be construed as a potential conflict of interest.

## Publisher's Note

All claims expressed in this article are solely those of the authors and do not necessarily represent those of their affiliated organizations, or those of the publisher, the editors and the reviewers. Any product that may be evaluated in this article, or claim that may be made by its manufacturer, is not guaranteed or endorsed by the publisher.
